# Improved grey water footprint model based on uncertainty analysis

**DOI:** 10.1038/s41598-023-34328-z

**Published:** 2023-05-02

**Authors:** Juan Li, Ma Lin, Yan Feng

**Affiliations:** 1 School of Hydraulic and Environmental Engineering, Changsha University of Science & Technology, Changsha, 410114 China; 2 Hunan Polytechnic of Water Resources and Electric Power, Changsha, 410114 China; 3 Engineering Research Center of Watershed Carbon Neutralization, Nanchang University, Ministry of Education, Nanchang, 330031 China

**Keywords:** Environmental sciences, Hydrology

## Abstract

In the practical water resources management, the allowable thresholds of pollutants are not unique. However, the conventional grey water footprint (GWF) model cannot deal with this uncertainty in the controlling threshold. To solve this problem, an improved GWF model and pollution risk evaluation method is designed according to the uncertainty analysis theory and maximum entropy principle. In this model, GWF is defined as the mathematical expectation of virtual water to dilute the pollution load within the allowable threshold, and the pollution risk is deduced by the stochastic probability by which GWF exceeds the local water resources. And then, the improved GWF model is applied in the pollution evaluation of Jiangxi Province, China. The results show that: (1) From 2013 to 2017, the annual GWF values of Jiangxi Province were 136.36 billion m^3^, 143.78 billion m^3^, 143.77 billion m^3^, 169.37 billion m^3^ and 103.36 billion m^3^, respectively. And their pollution risk values and grades were 0.30 (moderate), 0.27 (moderate), 0.19 (low), 0.22 (moderate), and 0.16 (low), respectively. In 2015, the determinant of the GWF was TP, and TN in other years. (2) The improved GWF model has an evaluation result which is basically consistent with WQQR, and it is an effective water resource evaluation method to deal with the uncertainty in controlling thresholds. (3) Compared with the conventional GWF model, the improved GWF model has better capacities in identifying pollution grades and recognizing pollution risks.

## Introduction

Freshwater with suitable quality is a basic requirement of human survival and social development^1^. However, with the rapid growth of economy, large amounts of contaminants are discharged into the natural aquatic environment, thereby seriously threatening the human health and the sustainable development of society^2^. Therefore, assessing the influences of pollution load on natural water resources is crucial.

In the current literature, grey water footprint (GWF) is the most commonly used tool to evaluate the impact of pollutants on aquatic environment^3^. To quantify the impact of pollutants on water resources, Mekonnen and Hoekstra^4^ proposed a GWF model based on virtual water theory. GWF is the amount of virtual water by which the pollution load is diluted below the allowable threshold^5^. GWF has advantages in simple calculation and intuitive results^6^. Moreover, the water quantity and quality are comprehensively considered^6^. Therefore, GWF has been widely used in water quality assessment in recent years. For example, Chapagain et al.^7^ and Mekonnen et al.^8^ applied GWF to the pollution of the global rice production and consumption. Liao et al.^9^ used GWF to analysis the interprovincial virtual grey water transfers for China’s final electricity demands. And Yan et al.^6^ evaluated the influences of noncarcinogenic heavy metals in mine wastewater of Dexing City, China.

Although GWF has been widely applied in the water resource management, the uncertainty of GWF needs to be further studied^10^. In the conventional GWF model, the basic hypothesis is that the water body receiving pollutants is considered definite, and the corresponding concentration limit is unique. However, through the comprehensive analysis of enormous agricultural GWF evaluation examples, Huang et al.^11^ found that in the areas with strong water system connectivity, the dilution water bodies and allowable thresholds are usually uncertain. Take the total phosphorus (TP) as an example, in the Chinese water resources management, the allowable threshold of TP in the farmland drainage channels which directly receives the agricultural sewage is 0.4 mg/L^12^. However, with the hydrological cycle, this phosphorus load will may enter the lake, in which the allowable threshold of TP is 0.01 mg/L or 0.05 mg/L^12^. As a result, the dilution water bodies and allowable thresholds of TP are not unique. Therefore, the uncertainty of GWF comes from the uncertainty of allowable limit caused by water system connectivity. And this problem exists whenever the allowable limits of different water bodies in the area are different. Nevertheless, the conventional GWF model has difficulties to deal with the uncertainties in allowable limits of pollutants, which limits its further application.

The objectives of this study are listed as follows: (1) design an improved GWF model according to the uncertainty analysis theory and maximum entropy principle; (2) evaluate the water resource shortage risk of Jiangxi Province by using the improved GWF; (3) discuss the effectiveness of GWF through a comparison with the water quality qualified rate (WQQR).

## Methods and materials

### Study area

**Figure 1:**
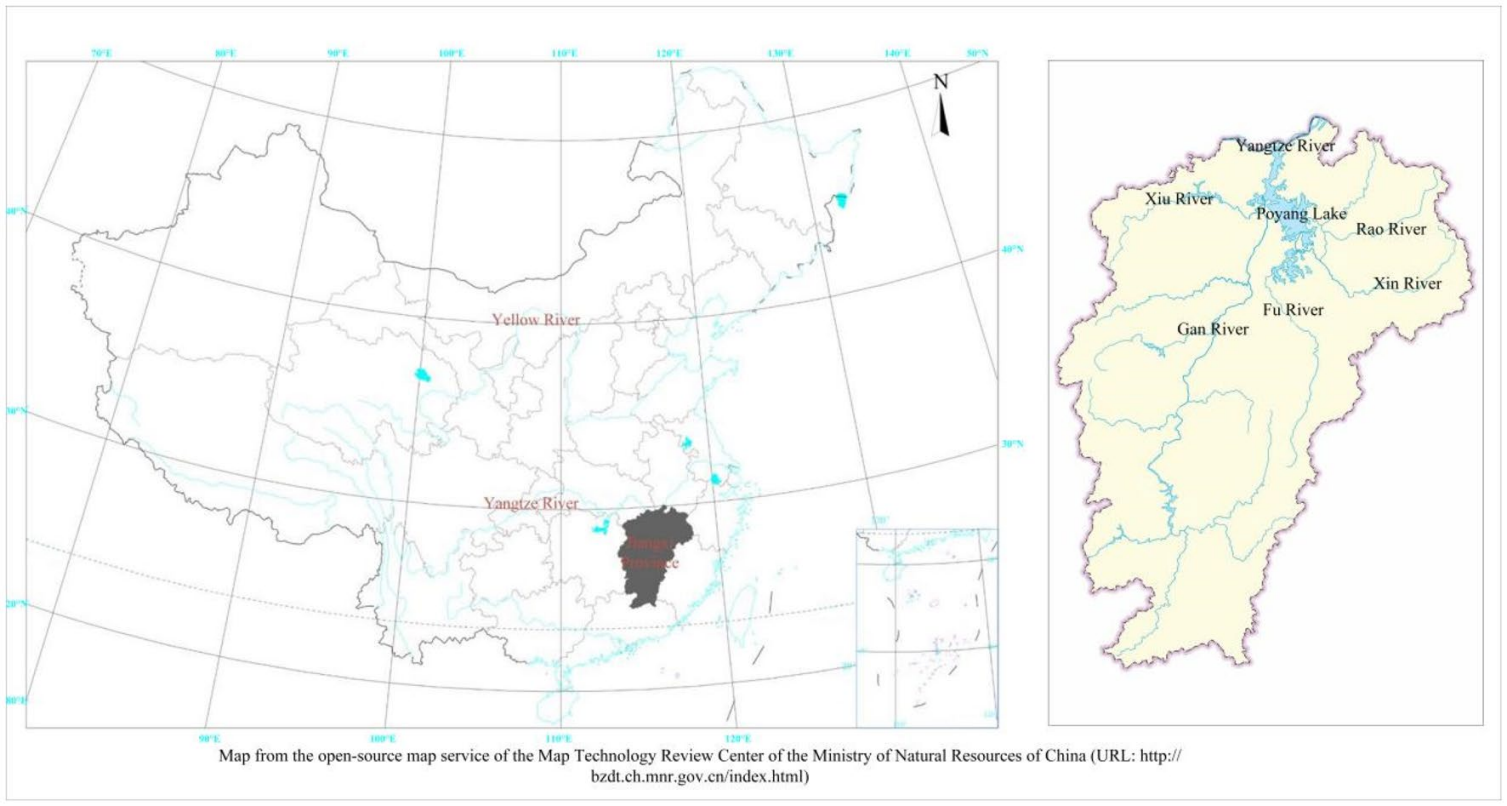
Study area.

As illustrated in Fig. 1, Jiangxi Province lies in the Poyang Lake Basin, the largest freshwater lake in China^13^. It is located in the central part of China with a land area of 166,900 km^2^ and a population of 4.65 million. Poyang Lake is an important habitat for migratory birds, finless porpoises, and other rare animals^14^. As a result, the protection of aquatic environment in Jiangxi Province is one of the priorities of water resource management in China.

Jiangxi Province is an important commodity grain and nonferrous metal production base in China^14^. In 2017, the gross regional product of Jiangxi Province reached 200.631 billion yuan, with the highest contribution rate of 48.1% in the secondary industry, followed by 42.7% in the tertiary industry, and the lowest contribution rate of 9.2% in the primary industry. Among them, Jiangxi Province is relatively developed in industry, contributing 38.9% to economic growth. However, with the rapid development of economy, a large number of pollutants are discharged into the natural water body, affecting significantly the local aquatic environment. Chemical oxygen demand, ammonia nitrogen, total nitrogen, and total phosphorus discharged into the water environment of Jiangxi Province respectively account for 5.08%, 4.14%, 3.73%, and 4.22% of the total national emissions^15^. Hu and Dai^13^ found that almost no eutrophication problem in Poyang Lake before the 1990s. However, as the pollution load into the lake increases sharply, Poyang Lake currently faces a moderate risk of eutrophication, posing a serious threat to migratory birds and finless porpoises^13^.

According to Hu and Dai^13^, the agriculture pollution can be represented by total nitrogen (TN), ammonia nitrogen (NH_3_-N), and total phosphorus (TP), and industrial pollution can be indicated by chemical oxygen demand (COD). Thus, these pollutants are used to evaluate the GWF in this study. In addition, the pollution load and water resource data are cited from the Chinese statistical yearbook published by Chinese National Bureau of Statistics^15^.

Jiangxi has a well-developed water system with a river network density of 0.11 km/km^2^, making it one of the provinces with the largest river network density in China^13^. However, the allowable limits of pollutants in these water bodies are quite different. According to the research of Hu and Dai^13^, the loosest and strictest controlling thresholds of pollutants are listed in Table 1.

**Table 1:** The loosest and strictest controlling thresholds of pollutants (mg/L).

The controlling thresholds of pollutants	TN	NH_3_-N	TP	COD
The loosest controlling threshold	2.0	2.0	0.4	40
The strictest controlling threshold	0.2	0.15	0.01	15

Due to the dense river networks, less water conservancy projects, and highly connected water systems, the migration and diffusion of pollutants in water environment are very strong, which makes it difficult to determine the specific dilution water body and allowable limit for each pollutant.

### Conventional GWF model

GWF is the amount of the virtual water, which dilutes the pollution load below the allowable limit. Its calculating formula is as follows^5,6^:

1 $$G_{n} = \frac{{M_{n} }}{{\left( {c_{n} - b_{n} } \right) \times 1000}},$$

where *G*_*n*_, *M*_*n*_, *c*_*n*_, and *b*_*n*_ are the GWF (billion m^3^), pollutant emission (t), allowable limit (mg/L), and background value (mg/L) of the *n*th pollutant, respectively^5,6^.

The water pollution level of the *n*th pollutant (*P*_*n*_) is as follows^5,6^:

2 $$P_{n} = \frac{{G_{n} }}{W},$$

where *W* is total water resources of the study area (billion m^3^).

*P*_*n*_ is a dimensionless index defined on [0, + ∞); it reflects the ratio of GWF of the *n*th pollutant to total water resources^5,6^. *P*_*n*_ > 1 indicates that the pollution load has exceeded the dilution capacity of the local water resources, thereby causing water shortage problem^5,6^.

In most cases, *G*_*n*_ and *P*_*n*_ of various pollutants are different. In the study of GWF, the regional aquatic environment quality is determined by the most severely polluted index; thus, the total GWF *G* and water pollution level *P* are defined as follows^5,6^:

3 $$\left\{ \begin{gathered} G = \max \{ G_{n} \} \hfill \\ P = \max \{ P_{n} \} \hfill \\ \end{gathered} \right.$$

*P* > 1 indicates that at least one pollutant has exceeded the dilution capacity of the local water resources, leading to water resource shortage^5,6^.

As shown in Eq. (1), a basic hypothesis of the conventional GWF is that the allowable limit parameter (*c*_*n*_) is definite and unique. However, as is introduced above, they are often uncertain in the natural hydrological cycle. In actual water resource management, the specific allowable limit *c*_*n*_ of the nth pollutant is difficult to determine, and only the upper and lower limits can be determined. Therefore, the conventional hypothesis is not suitable for evaluation the influences of pollution load on natural water resources and Eq. (1) cannot be used to directly evaluate the GWF of the study area.

### Improved GWF model

As introduced in Section "Conventional GWF model", a basic hypothesis of the conventional GWF is that the allowable limit parameter is definite and unique. However, they are often uncertain in the natural hydrological cycle. Therefore, Eq. (1) cannot be used to directly evaluate the GWF of pollutants. To solve this problem, an improved GWF model is designed in this section. Different from the conventional GWF, the hypothesis of the improved model is that the allowable limit is a random variable instead of a determined value.

This study regards the allowable limit *c*_*n*_ as a continuous random variable defined on [*l*_*n*_, *s*_*n*_], where *s*_*n*_ and *l*_*n*_ are the upper and lower values of *c*_*n*_, respectively. Denote the probability density function of *c*_*n*_ as *f* (*c*_*n*_). Based on the probability theory, *G*_*n*_ is also a random variable, and its mathematical expression is as follows:

4 $$\overline{{G_{n} }} = \int\limits_{ - \infty }^{ + \infty } {\left[ {\frac{{M_{n} }}{{\left( {c_{n} - b_{n} } \right) \times 1000}} \times f(c_{n} )} \right]dc_{n} }$$

As shown in Eq. (5), the risk probability *F*_*n*_ of water resource shortage induced by the pollutant is as follows:

5 $$F_{n} = \int\limits_{{c_{n} \in \Omega_{n} }} {f(c_{n} )} dc_{n} ,$$

and $$\Omega_{n} = \left\{ {c_{n} \left| {\frac{{M_{n} }}{{\left( {c_{n} - b_{n} } \right) \times 1000 \times W}} > 1} \right.} \right\}$$.

According to Eq. (3), the total risk probability of water resource scarcity in the study area is as follows:

6 $$F = \max \left\{ {F_{n} } \right\}$$

When the water function area samples are sufficient, statistical methods are used to deduce *f* (*c*_*n*_)^2,14^. Maximum entropy principle can be used to solve *f* (*c*_*n*_) when only the upper and lower limits *s*_*n*_ and *l*_*n*_ of *c*_*n*_ can be determined^2,14^. In uncertainty analysis, the uncertainty of random variables can be quantified by its entropy *H*_*n*_, as follows^2,14^:

7 $$H_{n} = \int\limits_{{c = l_{n} }}^{{s_{n} }} {\ln f(c_{n} ) \cdot f(c_{n} )} dc_{n}$$

Based on maximum entropy principle, the most probable *f* (*c*_*n*_) of the random variable *c*_*n*_ satisfies the following conditions^2,14,16^:

8 $$\begin{aligned} & \max :H_{n} = - \int\limits_{{b_{n} = l_{n} }}^{{s_{n} }} {f(c_{n} ) \cdot \ln f(c_{n} )dc_{n} } \\ & {\text{s.t.:}}\;\int\limits_{{c_{n} = l_{n} }}^{{s_{n} }} {f(c_{n} )dc_{n} } = 1 \\ \end{aligned}$$

According to functional analysis and optimization theory, it is easy to obtain:

9 $$f(c_{n} ) = \left\{ {\begin{array}{*{20}l} {\frac{1}{{s_{n} - l_{n} }}} \hfill & {l_{n} \le c_{n} \le s_{n} } \hfill \\ 0 \hfill & {{\text{otherwise}}} \hfill \\ \end{array} } \right.$$

In water resource management, the practical significance of Eq. (9) is that when only the upper and lower limits of allowable pollutant limit *c*_*n*_ can be determined, the most likely distribution of *c*_*n*_ is uniform distribution.

By taking Eq. (9) into Eqs. (4) and (5), we obtain the following:

10 $$\begin{aligned} \overline{{G_{n} }} & = \int\limits_{{l_{n} }}^{{s_{n} }} {\left[ {\frac{{M_{n} }}{{\left( {c_{n} - b_{n} } \right) \times 1000}} \times \frac{1}{{s_{n} - l_{n} }}} \right]dc_{n} } \\ & = \frac{{M_{n} }}{{(s_{n} - l_{n} ) \times 1000}} \times \ln \frac{{s_{n} - b_{n} }}{{l_{n} - b_{n} }} \\ \end{aligned}$$

and

11 $$F_{n} = \left\{ {\begin{array}{*{20}l} 0 \hfill & {\frac{{M_{n} }}{1000 \times W} + b_{n} < l_{n} } \hfill \\ {\frac{{M_{n} }}{{1000 \times W \times \left( {s_{n} - l_{n} } \right)}} + \frac{{b_{n} - l_{n} }}{{s_{n} - l_{n} }}} \hfill & {l_{n} \le \frac{{M_{n} }}{1000 \times W} + b_{n} \le s_{n} } \hfill \\ 1 \hfill & {s_{n} \le \frac{{M_{n} }}{1000 \times W} + b_{n} } \hfill \\ \end{array} } \right.$$

The water environmental quality is determined by the most serious pollution index; thus, $\overline{G}$ and *F* are defined as follows:

12 $$\left\{ \begin{gathered} \overline{G} = \max \{ \overline{G}_{n} \} \hfill \\ F = \max \{ F_{n} \} \hfill \\ \end{gathered} \right.$$

Mekonnen and Hoekstra^17^ suggested that the statistical error of pollution load is ± 20% probably. Therefore, on the basis of the *F* value, the water pollution is classified into 4 categories in this study, as follows:

1. Low (0 ≤ *F* ≤ 0.2), the risk of GWF exceeding total water resources is less than 20%, and the possibility of inducing water shortage is negligible.
2. Moderate (0.2 < *F* < 0.8), the risk of GWF exceeding the total water resources range from 20 to 80%, and the possibility of inducing water shortage is not negligible.
3. High (0.8 ≤ *F* ≤ 1.2), the risk of GWF exceeding total water resources is higher than 80%, and it can possibly induce water shortage.
4. Very high (*F* > 1.2), the GWF is higher than the total water resources; thus, it will certainly induce water shortages.

### Water quality qualified rate (WQQR)

WQQR is a water resource evaluation method proposed by China to adapt to different controlling objectives^13^. In this study, we use WQQR to test the validity of the improved GWF model. The calculation formula of WQQR is as follows^13^:

13 $$q = \frac{{m_{sat} }}{{m_{tot} }} \times 100{\text{\% }}$$

where *m*_*tot*_ is the amount of the total water quality monitoring sites, and *m*_*sat*_ is the amount of the sites whose water quality can satisfy its allowable limit.

In the water resource management of China, the water pollution is classified into three grades according to WQQR, as follows^13^:

1. Low (*q* ≥ 85%): the water environment is polluted slightly, and the water quality is basically at a natural level.
2. Moderate (55% ≤ *q* < 85%): the water environment is polluted moderately, and sometimes the water quality cannot be at a natural level.
3. High (*q* < 55%): the water environment is polluted seriously, and the water quality cannot be maintained at a natural level.

Currently, the WQQR model is mainly applied to China, while other countries have other water quality indexes, such as the Canadian Water Quality Index (CWQI) and Oregon Water Quality Index (OWQI)^18,19^. The study area of this study is Jiangxi Province, China, so the WQQR model is used to analyze water quality. If the study area is located in other countries, other water quality indicators can be used.

## Results and discussion

### Pollution load analysis

**Figure 2:**
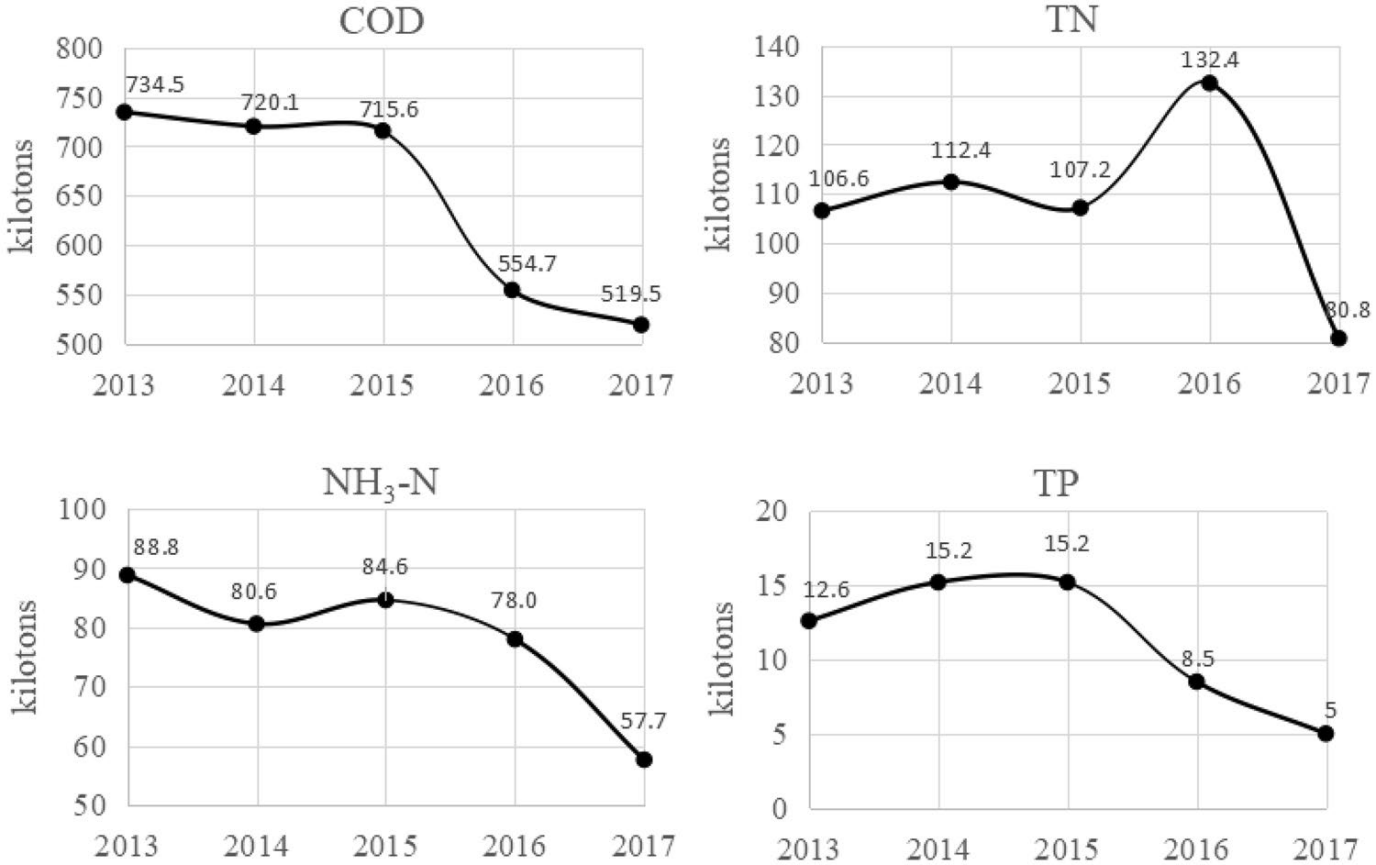
Pollution load of Jiangxi Province.

According to the Chinese statistical yearbook, the pollution load data of Jiangxi Province are listed in Fig. 2.

As illustrated in Fig. 2, from 2013 to 2017, the annual average value of the COD load discharged was 648,880 t, which was the largest of all. However, the overall trend declines annually, with 519,500 t in 2017, which was equivalent to 70.7% in 2013. The reason is that: the main source of COD is industrial wastewater discharge. Jiangxi Province has developed industries and dense industrial parks, so the annual average discharge of COD is the highest of the 4 pollutants. However, with the introduction of environmental protection policies in Jiangxi Province, supervision and management of enterprises have been strengthened, resulting in a decrease in COD emissions from enterprises.

The annual average value of TN load emitted was 107,880 t, which was the second largest. The value of TN fluctuated prior to 2016 and decreased rapidly after 2016. The maximum and minimum TN loads are 132,400 t in 2016 and 80,800 t in 2017, respectively. The main source of TN emissions in Jiangxi Province is agricultural non-point source pollution. Excessive use of fertilizers and pesticides may lead to an increase in TN emissions. Then with the improvement of sewage treatment facilities, the emission of TN decreases.

From 2013 to 2017, the average annual value of NH_3_-N load discharged was 77,940 t, which was equal to 72.2% of TN. The value of NH_3_-N fluctuated prior to 2015 and decreased rapidly after 2015. The maximum and minimum NH_3_-N loads are 88,800 t in 2013 and 57,700 t in 2017, respectively. The reason is that: Jiangxi Province has increased investment in sewage treatment facilities and improved its sewage treatment capacity, thereby reducing NH_3_-N emissions.

The pollution load of TP is the lowest among all pollutants. The average annual value of TP load emitted was 11,300 t, which was only 1.7% of COD. The value of TP increased slowly from 2013 to 2014 and decreased from 2015 to 2017. The maximum value of TP was 15,200 t in 2014 and 2015, and the minimum value was 5000 t in 2017, which is approximately 33% of the maximum value. TP in Jiangxi Province mainly comes from agricultural non-point source pollution. With the strengthening of water environment protection in Jiangxi Province, TP emissions have shown a downward trend since 2015.

### GWF evaluation results

**Figure 3:**
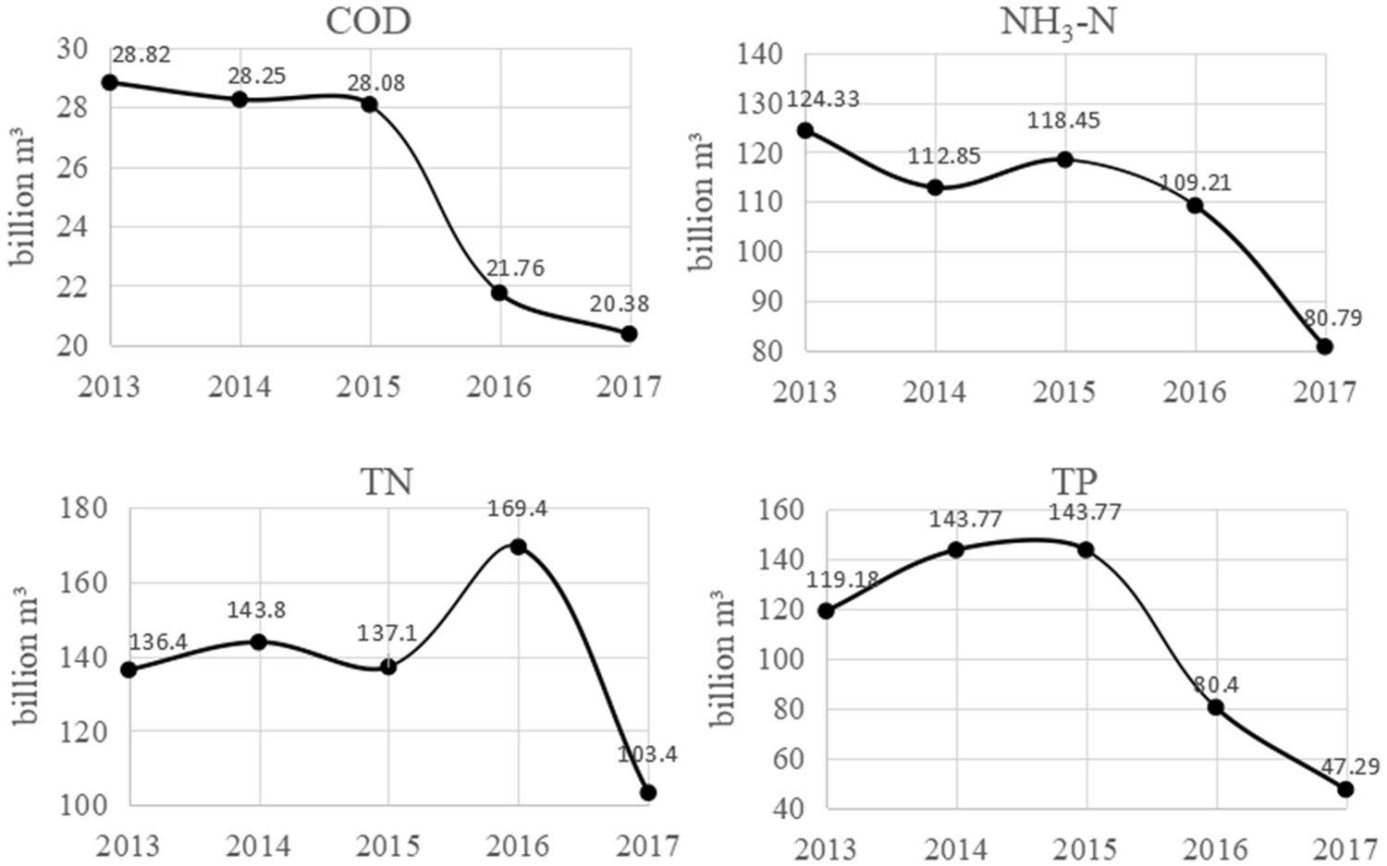
GWF evaluation results of pollutants.

According to Eq. (10), GWF and risk are generated and illustrated in Fig. 3.

As shown in Fig. 3, the GWF changes in the same trend as the pollution load. The value of COD showed a downward trend throughout the entire period. The value of TN indicated a fluctuating trend prior to 2016 and showed a rapid decline after 2016. The value of NH_3_-N indicated a fluctuating trend prior to 2015 and a downward trend after 2015. The value of TP showed a slow upward trend from 2013 to 2014 and a downward trend from 2015 to 2017.

The results show that GWF and pollutant discharge load are significantly different in value. In the entire period, the annual average GWF of COD, TN, and NH_3_-N are 25.46, 138.00, and 109.13 billion m^3^, respectively. Specially, although the TP discharge load is the lowest among all pollutants, its annual average GWF is 106.88 billion m^3^. In 2015, the GWF of TP was 143.77 billion m^3^, which is the largest of all pollutants.

The reason for this difference is that the GWF is affected by pollution load and allowable limits. Although the pollution load is low, it may also produce a large GWF for pollutants with low *c*_*n*_. For example, although the pollution load of COD is approximately 57.4 times of TP, its allowable limit is only 0.07% to 1% of TP. Thus, the GWF of TP is larger than COD.

### Pollution risk analysis of pollutants

**Figure 4:**
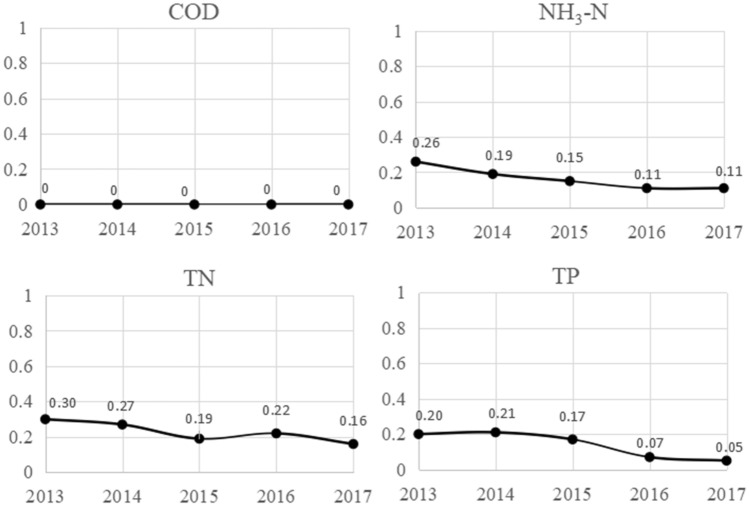
Pollution risk results of pollutants.

According to Eq. (11), the risk of each pollutant is generated and illustrated in Fig. 4.

As illustrated in Fig. 4, the change trend of pollution risk was different from the GWF and pollution load. From 2013 to 2017, the annual pollution risk of COD was zero, which was a “low” category. It indicated that its annual GWF was less than the total amount of water resources, and it was impossible to induce water shortages**.**

The pollution risk of NH3-N has been declining annually. Pollution risks were at the “low” level except for the moderate value in 2013. TN showed a downward trend in 2013–2015 and 2016–2017, but showed an upward trend in 2015–2016. Except for 2015 and 2017, which were at the “low” categories, the pollution risks of the remaining years were at the “moderate” categories.

From 2013 to 2014, the pollution risk of TP indicated an upward trend, and the annual risk index was more than 0.2, which was “moderate” grade. After 2014, the pollution risk of TP had a rapid declining trend, and the annual risk index was lower than 0.2, which was “low” grade.

The reason for this difference is that the pollution risk is influenced by not only the GWF, but also the total water resource. The greater value of *W* indicates lower pollution risk by the GWF. For example, as Figs. 1 and 3 show, though the GWF of NH_3_-N in 2015 was equal to 105% of 2014, *W* in 2015 was only 123% of 2014. Thus, the risk of NH_3_-N pollution in 2015 was lower than in 2014.

### Comprehensive evaluation results

**Figure 5:**
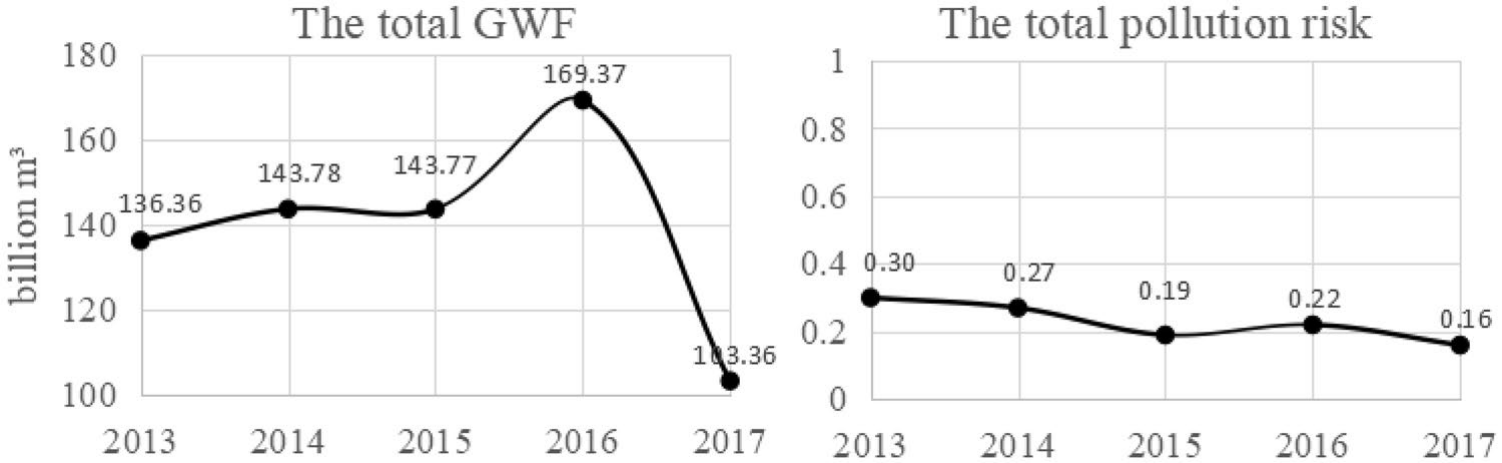
The total GWF and pollution risk.

According to Eq. (12), the total GWF and pollution risk are generated and illustrated in Fig. 5.

As listed in Fig. 5, the total GWF showed a slow upward trend from 2013 to 2016, and a rapid downward trend from 2016 to 2017. The maximum and the minimum values were 169.37 billion m^3^ in 2016 and 103.36 billion m^3^ in 2017, respectively. In 2015, the determinant of the GWF was TP, and TN in other years.

The change trend of the total pollution risk is inconsistent with the total GWF. It indicated a downward trend in 2013–2015 and 2016–2017 and an upward trend in 2015–2016. The maximum and the minimum values were obtained in 2013 and in 2017, respectively. The total pollution risk values in 2013, 2014, and 2016 ranged from 0.2 to 0.8, belonging to the “moderate” category. The values were lower than 0.2 and belonged to the “low” category in 2015 and 2017, which indicated that the possibility of inducing water shortage by pollution was negligible. The determinant of pollution risk was only TN unlike the GWF.

Combined with Figs. 3 and 4, the impact of various pollutants on water resources is ranked as follows: TN > TP > NH_3_-N > COD. TN and TP are indicative pollutants of agricultural nonpoint source pollution. Thus, to further improve the aquatic environment, strengthening the treatment of agricultural nonpoint source pollution is necessary.

### Effectiveness analysis of improved GWF

The evaluation results were compared with the calculated results of WQQR to verify the effectiveness of improved GWF. According to the water resources bulletin issued by Jiangxi water resources department, the evaluation results of WQQR are listed in Table 2.

**Table 2:** Comparison between the evaluation results of improved GWF and WQRR.

Year	Evaluation by WQQR		Evaluation by improved GWF		
	WQQR (%)	Pollution grade	GWF	Pollution risk	Pollution grade
2013	83.1	Moderate	136.36	0.30	Moderate
2014	83.6	Moderate	143.78	0.27	Moderate
2015	87.6	Low	143.77	0.19	Low
2016	85.3	Low	169.37	0.22	Moderate
2017	93.7	Low	103.36	0.16	Low

As shown in Table 2, from the perspective of pollution ranking, the improved GWF assessment result is consistent with WQQR, as follows: 2013 > 2014 > 2016 > 2015 > 2017.

From the perspective of the pollution grade, the improved GWF evaluation result is very close to WQQR. The difference only appeared in 2016. The grades of WQQR and the improved GWF were “low” and “moderate”, respectively. However, considering that the WQQR in 2016 was 85.3%, which was only 0.3% higher than the thresholds of “moderate” and “low”, this grade difference can be considered to be slight.

In order to further study whether there are differences in the assessment of pollution grades by different assessment methods, the Mann–Whitney rank sum test method in the nonparametric test is selected. This method requires a wide range of assumptions about the overall distribution of data, does not rely on the overall distribution form, and is not susceptible to extreme value perturbations. Therefore, it can better evaluate the differences between two sets of independent samples.

Based on Table 2, in this study, the pollution grades for 2013–2017 obtained by WQQR and improved GWF methods are defined as sample groups 1 and 2, respectively. Each sample group contains 5 pollution grade samples, sample group 1 contains 3 “low” and 2 “moderate,” and sample group 2 contains 2 “low” and 3 “moderate.” In order to facilitate the calculation, this study quantified the pollution grades “low”, “moderate” and “high” as 1, 2 and 3, respectively.

The data are entered into the SPSS 27 software, and after weighting the data for each classification, two independent samples from the Nonparametric Test are selected for testing. The test result is: Z = − 0.6, *P* = 0.690. According to the test level of α = 0.05 (*P* > 0.05), the test result shows that the difference is not statistically significant. This indicates that there is no difference in the evaluation effect between the WQQR and improved GWF methods.

Combined with the above discussion, it can be seen that the improved GWF has an evaluation result which is basically consistent with WQQR; and it is an effective water resource evaluation method to deal with the uncertainty in controlling thresholds.

### Comparison of improved and conventional GWF

In the research of GWF in China, when *c*_*n*_ in the study area is different, the drinking water standard is often used as the evaluation threshold^11^. According to the Chinese environmental quality standards for surface water, the controlling thresholds of COD, TN, NH_3_-N, and TP are 20, 1, 1, and 0.2 mg/L, respectively. Obviously, this drinking standard lies between the strictest ecological standard and loosest agricultural water quality standard.

According to Eqs. (1)–(3), the calculated results of conventional GWF are shown in Table 3.

**Table 3:** The evaluation results of the conventional GWF model.

Year	Water resources	GWF (billion m^3^)					Pollution risk				
	billion m^3^	COD	TN	NH_3_-N	TP	Total	COD	TN	NH_3_-N	TP	Total
2013	1424	367.25	1066	888	630	1066	0	0	0	0	0
2014	1631.8	360.05	1124	806	760	1124	0	0	0	0	0
2015	2001.2	357.8	1072	846	760	1072	0	0	0	0	0
2016	2221.1	277.35	1324	780	425	1324	0	0	0	0	0
2017	1655.1	259.75	808	577	250	808	0	0	0	0	0

Comparing Fig. 5 and Table 3, from the perspective of ranking, the calculation results of the traditional and the improved models are consistent. The GWF is ranked as follows: 2016 > 2014 > 2015 > 2013 > 2017; the comprehensive pollution level is ranked as follows: 2013 > 2014 > 2016 > 2015 > 2017.

The GWF value indicated a slight difference between them. As shown in Fig. 3 and Table 3, the GWF values of TN, NH_3_-N, and TP calculated by the conventional method are smaller than that of the improved method, but the COD is larger.

However, from the perspective of comprehensive pollution assessment, the results have evident differences. As shown in Table 3, according to the traditional model, the GWF in each year is less than the total amount of water resources (*W*). Thus, pollution-induced water shortage is impossible. Nevertheless, as is shown in Figs. 4 and 5, the improved model indicated a risk of GWF exceeding *W* in each evaluation year, and the risk of GWF exceeding *W* in 2013, 2014 and 2016 was higher than 20%. Moreover, the possibility of inducing water shortage cannot be neglected. According to Table 3, from 2013 to 2016, the annual WQQR of Jiangxi Province was lower than 100%. Therefore, the improved GWF method is more reasonable than the traditional method for identifying pollution grades.

The root cause of this difference is that the conventional methods consider the water resources of the study area as a whole for evaluation; thus, they neglect areas with the rigorous water quality targets, such as ecological protection zones. In natural water systems, the water quality control objectives of different regions are different. Even if the total pollution load is controlled within the allowable limit, the pollutants may still exceed the dilution capacity of the water body in some areas with strict water quality objectives. In the improved GWF, all possible water quality control objectives are considered comprehensively. Therefore, the impact of pollutants on areas with the rigorous water quality targets, such as ecological protection reserves, can be accurately reflected.

## Conclusion

From 2013 to 2017, the annual GWF values of Jiangxi Province were 136.36 billion m^3^, 143.78 billion m^3^, 143.77 billion m^3^, 169.37 billion m^3^ and 103.36 billion m^3^, respectively. And their pollution risk values and grades were 0.30 (moderate), 0.27 (moderate), 0.19 (low), 0.22 (moderate), and 0.16 (low), respectively. In 2015, the determinant of the GWF was TP, and TN in other years.

The improved GWF model has an evaluation result which is basically consistent with WQQR; and it is an effective water resource evaluation method to deal with the uncertainty in controlling thresholds. Furthermore, compared with the conventional GWF model, the improved GWF model has better capacities in identifying pollution grades and recognizing pollution risks.

## Data Availability

All data generated or analyzed during this study are included in this published article and in Fig. 2. Pollution load of Jiangxi Province of the article.
